# Drinking motivations in UK serving and ex-serving military personnel

**DOI:** 10.1093/occmed/kqaa003

**Published:** 2020-01-21

**Authors:** P Irizar, D Leightley, S Stevelink, R Rona, N Jones, K Gouni, J-A Puddephatt, N Fear, S Wessely, L Goodwin

**Affiliations:** 1 Department of Psychological Sciences, Institute of Psychology Health and Society, University of Liverpool, Liverpool, UK; 2 King’s Centre for Military Health Research, Institute of Psychiatry, Psychology & Neuroscience, King’s College London, London, UK; 3 Department of Psychological Medicine, Institute of Psychiatry, Psychology & Neuroscience, King’s College London, London, UK; 4 Academic Department of Military Mental Health, Institute of Psychiatry, Psychology & Neuroscience, King’s College London, London, UK

**Keywords:** Alcohol misuse, alcohol motivations, mental health, military personnel, quantitative methods

## Abstract

**Background:**

Drinking motivations within the UK military have not been studied despite the high prevalence of alcohol misuse in this group.

**Aims:**

We aimed to characterize drinking motivations and their demographic, military and mental health associations in UK serving and ex-serving personnel.

**Methods:**

Serving and ex-serving personnel reporting mental health, stress or emotional problems occurring in the last 3 years were selected from an existing cohort study. A semi-structured telephone interview survey examined participants’ mental health, help-seeking, alcohol use and drinking motivations.

**Results:**

Exploratory factor analysis of drinking motivations in military personnel (*n* = 1279; response rate = 84.6%) yielded 2 factors, labelled ‘drinking to cope’ and ‘social pressure’. Higher drinking to cope motivations were associated with probable anxiety (rate ratio [RR] = 1.4; 95% confidence interval [CI] = 1.3–1.5), depression (RR = 1.3; 95% CI = 1.2–1.4) and post-traumatic stress disorder (RR = 1.4; 95% CI = 1.3–1.6). Higher social pressure motivations were associated with probable anxiety (odds ratio = 1.1; 95% CI = 1.0–1.1). Alcohol misuse and binge drinking were associated with reporting higher drinking to cope motivations, drinking at home and drinking alone.

**Conclusions:**

Amongst military personnel with a stress, emotional or mental health problem, those who drink to cope with mental disorder symptoms or because of social pressure, in addition to those who drink at home or drink alone, are more likely to also drink excessively.

**Key learning points**

**What is already known about this subject:**

- Alcohol misuse is more prevalent in UK military personnel than the general population, with rates of help-seeking being lower than for other mental health problems.
- Alcohol misuse in military personnel may be associated with mental health, with alcohol commonly used as a coping mechanism to alleviate symptoms.
- Research from US military personnel has identified that drinking to cope motivations predict alcohol problems, yet no study has explored drinking motivations in a UK military sample.

**What this study adds:**

- This study identified ‘drinking to cope’ and ‘social pressure’ as the key drinking motivations in UK military personnel, with a self-reported stress, emotional or mental health problem.
- Probable depression, anxiety and post-traumatic stress disorder were associated with higher drinking to cope motivations, with anxiety having a weak relationship with social pressure motivations.
- Drinking to cope motivations were more common in individuals reporting alcohol misuse and binge drinking, with social pressure motivations also being associated with the latter. Personnel who drink at home and/or alone were also more likely to report harmful drinking behaviours.

**What impact this may have on practice or policy:**

- These findings can inform the development of tailored interventions for this high-risk occupational group, by identifying those at risk of drinking to cope and alcohol misuse, such as those with a mental health problem.
- Our study identified the need to integrate mental health and substance use services, particularly for military personnel, who may be drinking to cope with an existing mental health problem.
- We identified the importance of context in relation to alcohol harms, with personnel who drink at home or alone being more likely to report alcohol misuse and binge drinking.

## Introduction

Alcohol misuse is common in the UK Armed Forces (AF), at a higher prevalence than the general population [1]. Whilst rates of alcohol misuse are decreasing in both the UK general population and UK AF personnel, the rate of alcohol misuse remains high in the UK AF [2,3]. Moreover, help-seeking for alcohol problems is less common than for other mental health problems, amongst UK AF [4]. Given the levels of alcohol misuse and low recognition [5], it is important to understand the drinking motivations in UK AF personnel, and whether such motivations are associated with alcohol misuse.

Alcohol misuse is often co-morbid with mental health problems, particularly with post-traumatic stress disorder (PTSD) in the general population and among military personnel [6,7]. Common mental disorders (CMD), such as depression and anxiety, have a prevalence of ~20% among serving UK military personnel [8], double that of the general population working [9]. A relationship between CMD and alcohol misuse has been widely reported [10], with one possibility being that some people drink to reduce the distress associated with mental disorder symptoms [11].

Throughout history, alcohol has been integral to military culture by creating social bonds between personnel or for ‘de-stressing’ after deployment [12]. Recent research has begun exploring drinking motivations in US military personnel [13,14]. Motivations relating to enhancement (positive internal effects) and coping (to reduce negative affect) predicted alcohol use, with coping motives uniquely predicting alcohol-related problems after controlling for psychological distress [13]. US personnel, with and without PTSD, scored similarly on social, enhancement and conformity (‘fitting in’) drinking motivations, but those with PTSD were more likely to report drinking alcohol to cope [14].

No study to date has directly explored the drinking motivations in the UK AF. Therefore, we aimed to (i) identify drinking motivations among UK AF personnel; (ii) assess the association of demographic, military and mental health factors with drinking motivations and (iii) examine the association of drinking motivations and the context of drinking with alcohol misuse.

## Methods

Participants were identified from the third phase of the health and wellbeing cohort study of UK AF conducted by King’s Centre for Military Health Research between 2014 and 2016, which included data from existing members in previous phases [8], and a new sample who joined between August 2009 and March 2013 (*n* = 8093) [3]. The study sample is described in more detail elsewhere [4]. Participants who consented to future contact and answered ‘yes’ to the question, ‘have you had a mental health, stress or emotional problem in the past three years?’, during the third phase of the cohort study, were invited to participate in a semi-structured telephone interview study designed to explore help-seeking [4], which also provided data for the current study.

Of the 2017 participants who self-reported a mental health, stress or emotional problem, 1714 were randomly selected (85%) to participate in a telephone interview, with a total of 1450 interviews completed (response rate 85%) [4]. Of these completed interviews, 171 were excluded as they had taken part in a preliminary phase of the cohort study that did not use a random selection of participants, and so, 1279 interviews were used for the subsequent analyses. The procedure for the present study has been described in more detail elsewhere [4]. Ethical approval was granted by the UK Ministry of Defence Research Ethics Committee (ref: 535/MODREC/14) and King’s College London Psychiatry Nursing and Midwifery Research Ethics Subcommittee (ref: PNM/12/13-169).

The Alcohol Use Disorder Identification Test-Consumption (AUDIT-C) [15] uses three questions to measure frequency of alcohol use (responses range from ‘never’ to ‘four or more times a week’), units consumed on a typical day of drinking (ranging from ‘1 or 2’ to ‘≥10’) and binge drinking (i.e. 6+ units on one occasion; ranging from ‘never’ to ‘daily or almost daily’). Scores for each question ranged from 0 to 4, with scores of 0 reflecting no alcohol use. Overall scores ranged from 0 to 12, with a high cut-off of ≥10 being used to indicate alcohol misuse, due to the high prevalence among UK military personnel [16].

Drinking motivations were measured using selected items taken from the Hilton Drinking Behaviour Questionnaire (HDBQ) [17] and Drinking Motives Questionnaire-Revised (DMQ-R) [18]. The five items measuring drinking motivations were selected from the HDBQ: how often participants drink to cope with distressing or disturbing thoughts, because of loneliness, to escape troubles, to forget the past and to put them at ease with other people. The remaining 28 items were excluded as they measured drinking behaviours (e.g. have you ever been violent after drinking). Due to study constraints on the length of the interview, particular items from the DMQ-R were excluded which seemed more relevant for an adolescent population (e.g. so that others will not kid you about not drinking), and so, 7 items out of 20 were selected. These included how often participants drink because of pressure from friends, to be sociable, to help when they feel depressed/nervous, to cheer them up, to get drunk, to fit in with a group and so they do not feel left out. A five-point Likert scale ranging from ‘never’ to ‘always’ was used.

Anxiety was measured using the seven-item Generalised Anxiety Disorder assessment (GAD-7) [19]. Scores of 10 were used to indicate probable anxiety (scores range from 0 to 21) [19]. The nine-item Patient Health Questionnaire (PHQ-9) [20] assessed depression, with scores of ≥15 (scores range from 0 to 27) indicating probable depression, based upon the recommended higher threshold [20]. PTSD was measured using the 20-item self-report PTSD Checklist (PCL-5) [21]. Scores of ≥38 (scores range from 0 to 80) indicated probable PTSD [21].

Military characteristics were measured by asking participants their service, rank, engagement, serving status, if they had been deployed to either Afghanistan or Iraq and if so, which was their last deployment. Participants were asked where they usually drink—the choices being home, military mess hall, public drinking establishments, civilian friends’ homes, military friends’ homes and others. They were asked who they usually drink with—military friends, civilian friends, family, spouse/partner, alone and other; and finally, the context in which, and with whom, they drank the most alcohol in a single session.

The descriptive demographic, military and mental health characteristics of participants were outlined using frequencies and percentages. (i) Exploratory factor analysis (EFA) examined the factor structure of the drinking motivations, using the principal factors method as the data violates the assumption of multivariate normality, with Promax (oblique) rotation to allow the factors to correlate. It was decided *a priori* that items with a factor loading of <0.40 would be excluded. Parallel analysis, a scree plot and Kaiser’s rule were used to determine the number of factors to retain.

The distribution and dispersion of the drinking motivations (derived from the factor analysis) were assessed using a Chi-Square goodness of fit test. (ii) Negative binomial and Poisson regression models explored the association between drinking motivations as the outcome and demographics (age, sex and marital status), military (rank, engagement, service, serving status and deployment) and mental health factors (GAD-7, PHQ-9 and PCL-5). (iii) Logistic regressions explored whether drinking motivations or the context of drinking were associated with alcohol misuse, using AUDIT-C caseness, and a subcomponent of the AUDIT-C, representing binge drinking. Adjustment was carried out for demographic, military and mental health variables which were significantly associated (*P* < 0.05) with the outcome, as well as sex and age.

Response weights were applied to account for non-response, based on variables associated with responding (i.e. age, rank and service) [4]. Unweighted frequencies, weighted percentages, weighted medians with interquartile ranges (25–75%), weighted rate ratios, odds ratios and 95% confidence intervals are reported. Statistical significance was defined as *P* < 0.05. All analyses were conducted with survey (svy) commands to account for weighting, using the computer software Stata/SE 15 (StataCorp., College Station, TX, USA).

## Results

The sample (*n* = 1279) included 707 serving (55%) and 572 ex-serving (45%) military personnel. Most were male (84%), married or in a long-term relationship (80%), in the Army (66%), and serving or had served as a regular (80%). The weighted mean (±SD) age was 41.1 (±9.6), ranging from 20.6 to 68.3 years. A total of 25% had not been deployed to either Iraq or Afghanistan, 20% reported deployment to Afghanistan, 25% to Iraq and 31% had deployed to both operations. About 26% were commissioned officers, 60% were non- commissioned officers and 14% held other ranks (private soldier equivalent). About 18% scored above the validated cut-offs for probable anxiety, 8% for probable depression and 8% for probable PTSD and 18% met the criteria for alcohol misuse (Supplementary Table 1, available at *Occupational Medicine* Online). Participants who reported abstinence (*n* = 91) were excluded from all analyses (total *n* = 1188).

The findings of the EFA are summarized in Table 1. The scree plot indicated a two-factor solution and the parallel analysis recommended a four-factor solution. However, no items had a factor loading of 0.40 or above on the third or fourth factors. Kaiser’s criterion recommends dropping components with an eigenvalue <1.0; the eigenvalues for the third and fourth factors were 0.24 and 0.11, respectively. Furthermore, the third and fourth factors only explained an additional 3% of variance, combined. Taken together, a two-factor model was deemed the most appropriate.

**Table 1.** Results of the exploratory factor analysis on selected items of the HDBQ and DMQ-R^a,b^ (*n* = 1188)

Factor	Item	Original scale	Factor loading (Promax)
Drinking to cope	To help you cope with distressing or disturbing thoughts	HDBQ	0.76
	Because of loneliness	HDBQ	0.63
	To escape from your troubles	HDBQ	0.83
	To forget the past	HDBQ	0.70
	Because it helps when you feel depressed or nervous	DMQ-R	0.81
	To cheer you up when you are in a bad mood	DMQ-R	0.64
	To get drunk	DMQ-R	0.42
	Eigenvalue		3.71
	% Variance		31%
Social pressure	Because your friends put pressure on you to drink	DMQ-R	0.51
	To fit in with a group	DMQ-R	0.79
	So you will not feel left out	DMQ-R	0.78
	To be sociable	DMQ-R	0.41
	To put you at ease with other people	HDBQ	0.41
	Eigenvalue		1.70
	% Variance		14%
	Total % variance		45%

^a^HDBQ, Hilton Drinking Behaviour Questionnaire [17]; DMQ-R, Drinking Motives Questionnaire-Revised [18].

^b^Items with factor loadings <0.40 were excluded.

Factor 1 was labelled ‘drinking to cope’ and included seven items, all of which had positive loadings. Factor 2 was labelled ‘social pressures’ and included five items, all with positive loadings. The Kaiser-Meyer-Olkin measure of sampling adequacy (0.86) indicates a low proportion of common variance among the set of variables, suggesting that the data were well-suited for factor analysis. Cronbach’s alpha for drinking to cope (*α* = 0.84) indicated good internal consistency and for social pressures (*α* = 0.69), approached the cut-off for acceptability (i.e. 0.70). Drinking to cope and social pressures were significantly correlated (*r* = 0.25, *P* < 0.001).

Due to the over-dispersion of drinking to cope motivations, a negative binomial regression model explored associations (Table 2). Personnel meeting the criteria for probable anxiety, depression and PTSD reported higher drinking to cope motivations than those without a mental health problem, remaining significant after adjustment. Throughout all adjustments, being single and having a former relationship (relative to being married or in a long-term relationship) were associated with higher drinking to cope motivations. Older personnel (≥50), compared to younger personnel (i.e. 20–35) and Royal Air Force personnel, compared to Army personnel were less likely to report drinking to cope motivations. However, the relationship between being in the Royal Air Force and drinking to cope was weak.

**Table 2.** Negative binomial regression analysis indicating the risk of drinking to cope motivations for alcohol consumption (outcome variable) with demographic, military and mental health associations (explanatory variable)

Variable	Drinking to cope		
	Median (IQR 25–75%)	Unadjusted RR (95% CI)	Adjusted RR (95% CI)
Sex			
Male	10.00 (8.00–12.00)	1	1
Female	10.00 (8.00–12.00)	0.96 (0.91–1.02)	0.95 (0.90–1.00)
Age			
20–35	11.00 (8.00–13.00)	1	1
36–49	10.00 (8.00–12.00)	0.92 (0.85–0.99)*	0.95 (0.89–1.01)
≥50	9.00 (7.00–11.00)	0.85 (0.78–0.92)***	0.87 (0.80–0.94)**
Marital status			
Married or long-term relationship	10.00 (8.00–12.00)	1	1
Single	11.00 (9.00–14.00)	1.15 (1.08–1.24)***	1.10 (1.04–1.16)**
Former relationship	10.00 (8.00–15.00)	1.18 (1.07–1.30)**	1.13 (1.04–1.22)**
Service			
Army	10.00 (8.00–12.00)	1	1
Naval Services	9.00 (8.00–12.00)	0.99 (0.88–1.12)	1.00 (0.94–1.08)
Royal Air Force	9.00 (8.00–11.00)	0.93 (0.86–1.01)	0.95 (0.91–1.00)*
Rank			
Commissioned Officer	9.00 (8.00–11.00)	1	1
Non-commissioned Officer	10.00 (8.00–12.00)	1.08 (1.02–1.14)**	1.03 (0.99–1.07)
Other ranks	11.00 (9.00–14.00)	1.24 (1.12–1.37)***	1.10 (1.01–1.20)*
Engagement			
Regular	10.00 (8.00–12.00)	1	1
Reservist	10.00 (8.00–12.00)	1.00 (0.94–1.06)	1.00 (0.95–1.06)
Serving status			
Serving	10.00 (8.00–12.00)	1	1
Discharged	10.00 (8.00–12.00)	1.01 (0.96–1.07)	1.03 (0.98–1.07)
Deployment			
Not deployed	9.00 (8.00–12.00)	1	1
Afghanistan	10.00 (8.00–13.00)	1.11 (1.04–1.20)**	1.06 (1.00–1.12)
Iraq	10.00 (8.00–12.00)	1.03 (0.96–1.10)	1.03 (0.97–1.09)
Both	10.00 (8.00–12.00)	1.01 (0.95–1.07)	1.01 (0.95–1.07)
Mental health^a^			
Anxiety non-case	9.00 (8.00–11.00)	1	1
Anxiety case	12.00 (9.00–17.00)	1.38 (1.27–1.50)***	1.35 (1.25–1.46)***
PTSD non-case	10.00 (8.00–12.00)	1	1
PTSD case	14.50 (11.00–21.00)	1.56 (1.41–1.73)***	1.30 (1.20–1.41)***
Depression non-case	9.00 (8.00–12.00)	1	1
Depression case	16.00 (11.00–21.00)	1.64 (1.44–1.86)***	1.43 (1.28–1.60)***

Median units and interquartile ranges (IQRs) are shown. Unadjusted and adjusted^b^ values are presented, with rate ratio (RR) and 95% confidence intervals (CIs) (*n* = 1188).

^a^General Anxiety Disorder (GAD-7) identifies probable anxiety; Patient Health Questionnaire (PHQ-9) identifies probable depression; the PTSD Checklist (PCL-5) identifies probable post-traumatic stress disorder.

^b^Adjusted for age, sex, marital status, serving status, engagement, deployment, GAD-7, PHQ-9 and PCL-5 (did not adjust mental health variables for co-morbid mental health caseness).

*P < 0.05, **P < 0.01, ***P < 0.001.

Given the non-normal distribution of social pressure motivations, Poisson regressions were used to examine associations (Table 3). After adjustment, there was a weak association between meeting the criteria for probable anxiety and higher social pressure drinking motivations. Throughout all adjustments, those who were older (i.e. ≥50) reported lower social pressure motivations than those who were aged 20–35 years. Relative to those who were married or in a long-term relationship, personnel who had a former relationship (i.e. divorced or widowed) reported lower social pressure motivations, still remaining after adjustments, though the relationship was weak. Personnel who had left service reported lower social pressure motivations than those who were still serving.

**Table 3.** Poisson regression analysis indicating the risk of social pressure motivations for alcohol consumption (outcome variable) with demographic, military and mental health associations (explanatory variable)

Variable	Social pressure		
	Median (IQR 25–75%)	Unadjusted RR (95% CI)	Adjusted RR (95% CI)
Sex			
Male	8.00 (7.00–10.00)	1	1
Female	8.00 (7.00–10.00)	1.00 (0.97–1.04)	0.96 (0.92–1.01)
Age			
20–35	9.00 (7.00–11.00)	1	1
36–49	8.00 (7.00–10.00)	0.95 (0.90–0.99)*	0.96 (0.92–1.01)
≥50	8.00 (6.00–9.00)	0.85 (0.81–0.90)***	0.89 (0.84–0.95)**
Marital status			
Married or long-term relationship	8.00 (7.00–10.00)	1	1
Single	9.00 (7.00–10.00)	1.05 (1.00–1.1)	1.04 (0.98–1.10)
Former relationship	8.00 (6.00–9.00)	0.94 (0.90–0.99)*	0.95 (0.91–0.99)*
Service			
Army	8.00 (7.00–10.00)	1	1
Naval Services	8.00 (7.00–10.00)	1.02 (0.94–1.10)	1.02 (0.96–1.09)
Royal Air Force	9.00 (7.00–10.50)	1.00 (0.95–1.06)	1.01 (0.98–1.04)
Rank			
Commissioned Officer	9.00 (7.00–10.00)	1	1
Non-commissioned Officer	8.00 (7.00–10.00)	0.98 (0.92–1.05)	0.97 (0.94–1.00)
Other ranks	8.00 (7.00–10.00)	0.99 (0.90–1.08)	0.96 (0.87–1.04)
Engagement			
Regular	9.00 (7.00–10.00)	1	1
Reservist	8.00 (6.50–10.00)	0.95 (0.91–1.00)	0.98 (0.94–1.02)
Serving status			
Serving	9.00 (7.00–11.00)	1	1
Discharged	8.00 (6.00–10.00)	0.91 (0.87–0.94)***	0.94 (0.90–0.98)***
Deployment			
Not deployed	8.00 (7.00–10.00)	1	1
Afghanistan	9.00 (7.00–11.00)	1.08 (1.02–1.16)*	1.04 (0.98–1.10)
Iraq	8.00 (6.00–10.00)	1.00 (0.96–1.05)	1.02 (0.98–1.07)
Both	9.00 (7.00–10.00)	1.07 (1.02–1.12)**	1.04 (0.99–1.10)
Mental health^a^			
Anxiety non-case	8.00 (7.00–10.00)	1	1
Anxiety case	9.00 (7.00–11.00)	1.04 (0.99–1.10)	1.05 (1.00–1.11)*
PTSD non-case	8.00 (7.00–10.00)	1	1
PTSD case	9.00 (7.00–11.00)	1.04 (0.96–1.11)	1.05 (0.98–1.13)
Depression non-case	8.00 (7.00–10.00)	1	1
Depression case	9.00 (7.00–11.00)	1.07 (0.98–1.17)	1.09 (1.00–1.19)

Median units and interquartile ranges (IQRs) are shown. Unadjusted and adjusted^b^ values are presented, with rate ratio (RR) and 95% confidence intervals (CIs) (*n* = 1188).

^a^General Anxiety Disorder (GAD-7) identifies probable anxiety; Patient Health Questionnaire (PHQ-9) identifies probable depression; the PTSD Checklist (PCL-5) identifies probable post-traumatic stress disorder.

^b^Adjusted for age, sex, marital status, engagement, service and deployment.

*P < 0.05, **P < 0.01, ***P < 0.001.

Personnel who met the criteria for alcohol misuse reported higher drinking to cope motivations than those who did not. After adjustment, personnel who met the criteria for alcohol misuse also reported higher social pressure motivations. Meeting the criteria for alcohol misuse was associated with drinking at home (relative to all other drinking locations) and drinking alone (relative to drinking with military friends) (Table 4). Personnel who reported frequent binge drinking reported higher drinking to cope and social pressure motivations, remaining significant after adjustment. Drinking at home (relative to all other locations), drinking alone and drinking with a spouse or partner (relative to drinking with military friends) were associated with significantly higher odds of frequent binge drinking (Table 5).

**Table 4.** Logistic regression analyses exploring the risk of alcohol misuse (AUDIT-C; outcome variable) with motivations for drinking and context of drinking associations (explanatory variables)

Variable	AUDIT-C case (*n* = 236)^a^			
	*n*	%	Unadjusted OR (95% CI)	Adjusted OR (95% CI)
Drinking motivations				
Drinking to cope^b^	13.00 (median)	IQR 11.00–18.00	1.26 (1.21–1.32)***	1.26 (1.21–1.32)***
Social pressure	9.00 (median)	IQR 7.00–10.00	1.03 (1.00–1.07)	1.04 (1.00–1.09)*
Drinking location				
Home	117	32.41	1	1
Mess	25	12.32	0.29 (0.17–0.49)***	0.32 (0.19–0.54)***
Pubs	68	17.04	0.43 (0.31–0.60)***	0.38 (0.27–0.55)***
Civilian friends’ homes	7	6.54	0.15 (0.06–0.36)***	0.15 (0.06–0.35)***
Military friends’ homes	9	14.52	0.35 (0.19–0.67)**	0.35 (0.17–0.70)**
Other	10	18.18	0.46 (0.23–0.94)*	0.47 (0.21–1.01)
Drinking with				
Military friends	65	15.66	1	1
Other	8	15.69	1.00 (0.41–2.45)	0.94 (0.34–2.58)
Civilian friends	46	14.70	0.93 (0.63–1.37)	0.81 (0.50–1.31)
Spouse/partner	57	23.55	1.66 (1.14–2.42)*	1.60 (0.99–2.59)
Family	12	18.75	1.24 (0.56–2.78)	1.15 (0.46–2.84)
Alone	48	47.52	4.88 (3.04–7.83)***	4.64 (2.55–8.42)***

Row frequencies and percentages are shown. Unadjusted and adjusted odds ratios (ORs) and 95% confidence intervals (CIs) are presented (*n* = 1188).^c,d^

^a^Reference group = AUDIT-C non-case (

= 951).

^b^Medians are weighted.

^c^Adjusted for age, sex, service, serving, GAD-7, PHQ-9 and PCL-5.

^d^Reference categories are most common group.

*P < 0.05, **P < 0.01, ***P < 0.001.

**Table 5.** Logistic regression analyses exploring the risk of binge drinking (outcome variable) with motivations for drinking and context of drinking associations (explanatory variables)

Variable	Binge drink weekly or more (*n* = 428)^a^			
	*n*	%	Unadjusted OR (95% CI)	Adjusted OR (95% CI)
Drinking motivations				
Drinking to cope (median IQR)^b^	11.00	(9.00–15.00)	1.24 (1.19–1.30)***	1.27 (1.21–1.34)***
Social pressure (median IQR)	9.00	(7.00–11.00)	1.06 (1.02–1.11)**	1.06 (1.01–1.11)*
Drinking location				
Home	183	50.69	1	1
Mess	58	28.57	0.39 (0.24–0.62)***	0.40 (0.25–0.64)**
Pubs	126	31.58	0.45 (0.33–0.60)***	0.46 (0.33–0.58)***
Civilian friends’ homes	23	21.50	0.27 (0.15–0.48)***	0.28 (0.15–0.51)***
Military friends’ homes	22	35.48	0.53 (0.31–0.91)*	0.53 (0.30–0.92)*
Other	16	29.09	0.40 (0.24–0.67)**	0.39 (0.24–0.67)**
Drinking with				
Military friends	126	30.36	1	1
Other	15	29.41	0.96 (0.55–1.66)	0.94 (0.52–1.70)
Civilian friends	99	31.63	1.06 (0.82–1.37)	1.08 (0.82–1.43)
Spouse/partner	110	45.45	1.91 (1.43–2.56)***	1.95 (1.45–2.64)***
Family	16	25.00	0.76 (0.39–1.48)	0.75 (0.39–1.46)
Alone	62	61.39	3.65 (2.54–5.23)***	3.55 (2.36–5.32)***

Row frequencies and percentages are shown. Unadjusted and adjusted odds ratios (ORs) and 95% confidence intervals (CIs) are presented (*n* = 1188).^c,d^

^a^Reference group = binge drink less than weekly (*n* = 759).

^b^Medians are weighted.

^c^Adjusted for age, sex, engagement and GAD-7.

^d^Reference categories are most common group.

*P < 0.05, **P < 0.01, ***P < 0.001.

## Discussion

The principal findings of this study were that UK military personnel who met the criteria for alcohol misuse and engaged in frequent binge drinking reported higher drinking to cope motivations. Those with mental disorder symptoms were more likely to report higher drinking to cope. There was a weak association between anxiety and higher social pressure motivations. Those of a younger age reported higher drinking to cope and social pressure motivations. Personnel holding lower ranks and not in an intimate relationship were more likely to report drinking to cope. Still serving in the military was also a risk for drinking due to social pressure. Drinking at home and/or alone was associated with meeting the criteria for alcohol misuse and frequent binge drinking and drinking with a partner was associated with the latter.

One strength of this study is the large sample of UK personnel, with a high response rate (85%). We controlled for a wide range of covariates to determine the influence of possible confounders and used well-validated measures of mental health and alcohol misuse [15,19–21]. We know from the previous rounds of the cohort study that mental health status did not predict response [3]. However, alcohol misuse was associated with non-response in the phase 3 sample [3]. Furthermore, although the sample size was sufficient, some of the associations were based on a small number of respondents, particularly for the context of drinking. Due to the nature of self-report, there may have been biases in reporting, given that interviews focussed on sensitive topics, with recall bias also being a concern with alcohol consumption. Furthermore, the sample was selected based on having a subjective mental health, stress or emotional problem in the last 3 years, so the findings are only representative of personnel who acknowledge this type of issue.

The identified drinking motivations, drinking to cope and social pressure—and their associations with alcohol problems—are similar to those observed in studies of both US AF and the UK general population [13,14]. In US AF, problem drinking was associated with coping motives, even after controlling for distress, with coping motives mediating the relationship between PTSD severity and alcohol-related harm [13,14]. In a comparison of drinking motivations in veterans and students, both reported social motives relating to binge drinking and alcohol misuse, but coping motives were associated with problem drinking in veterans only [22]. We identified a correlation between drinking to cope and social pressure motives, suggesting that those reporting high drinking to cope motives also report higher social pressure motives. The odds of meeting the criteria for alcohol misuse and binge drinking appear to increase when drinking is driven by drinking to cope, with a weak relationship with social pressure motives.

One explanation for the relationship between coping motivations and alcohol misuse is the self-medication hypothesis [23], whereby individuals drink to offset psychological symptoms. Within civilian samples, drinking to cope motives moderate the relationship between alcohol misuse and mental health problems [24]. Our study outcomes contribute to existing literature, demonstrating associations between mental health, drinking to cope motives and alcohol misuse, in a military sample. Military personnel may use alcohol to cope with stress or symptoms of a mental health problem. Interestingly, personnel with probable anxiety reported higher social pressure motives, after adjustments. Personnel with higher anxiety may be more vulnerable to social pressures if they are more likely to worry about how they are viewed by peers.

In our study, certain sociodemographic characteristics were associated with reporting higher drinking to cope motives, such as younger age, holding a lower rank and being single. Previous US and UK AF research studies confirm that these are important associates of alcohol misuse [1,25]. We observed that younger personnel and those still serving in the military reported higher social pressure motives. Military culture has a role in facilitating heavy drinking, through inconsistent drinking policies, and work environments that encourage drinking, such as breaks in operational deployment [26]. Personnel who may be more likely to embrace the military culture or be readily influenced by it, such as young people, might misuse alcohol due to social pressures. However, the association between social pressure motivations and alcohol misuse/binge drinking was weak.

Drinking alone is a predictor of alcohol problems in the general population [27]. We extend these findings to a military population, showing that personnel who drank at home and/or alone were more likely to meet the criteria for both alcohol misuse and binge drinking. Individuals with mental health problems tend to drink alone, but consequently, drinking alone increases the risk of harmful drinking [28]. We may have expected the associations to attenuate after adjusting for mental health, yet they remained significant, possibly suggesting that drinking alone/at home is associated with alcohol misuse, regardless of mental health. Drinking with a partner was also associated with binge drinking, which may be because individuals are attracted to others with similar drinking habits, or seek out others with congruent drinking behaviours [29].

Our study shows the significance of context in relation to alcohol harm, identifying that personnel who drink at home, alone or with a partner, may be particularly at risk. Interventions that assist with self-monitoring of alcohol consumption, such as smartphone applications [30], may be effective for personnel who drink at home/alone, where the volume of alcohol may be underestimated. Mental health problems may drive drinking to cope, whereby military-affiliated personnel use alcohol to alleviate negative affect. Those who drink to cope with mental disorder symptoms are at heightened risk of alcohol misuse. There is a need for the integration of mental health and substance use services—mental health treatments should be provided in parallel with alcohol treatments, as individuals with mental health problems may use alcohol to cope.

## Funding

The research was funded by the Medical Research Council (MR/N028244/2).

## Competing interests

S.W. and S.S. are affiliated to the National Institute for Health Research (NIHR) Biomedical Research Centre at South London and Maudsley NHS Foundation Trust and King’s College London. S.W. is a trustee of the charity Combat Stress. The views expressed are those of the author(s) and not necessarily those of the NHS, the NIHR or the Department of Health and Social Care. N.J. is a serving member of the British Army seconded to King’s College London. The remaining authors declare no declarations of interests.

## Supplementary Material

kqaa003_suppl_Supplementary-Table-1
