# Supplementary material for: Drinking motivations in UK serving and ex-serving military personnel
Source: Occup Med (Lond). 2020 Jan 21;70(4):259–67. doi: 10.1093/occmed/kqaa003 (PMC7305700; doi:10.1093/occmed/kqaa003)
Supplement: kqaa003_suppl_Supplementary-Table-1 [file kqaa003_suppl_supplementary-table-1.doc]

**Supplementary Table 1**

**Sample characteristics of 1,279 serving and ex-serving military personnel.**

| Characteristic | | Total ^a^ | *N* | Weighted column % |
| --- | --- | --- | --- | --- |
| Sex | | 1279 |  |  |
|  | Male |  | 1079 | 84 |
|  | Female |  | 200 | 15 |
| Age | | 1279 |  |  |
|  | 20 - 35 |  | 402 | 31 |
|  | 36 - 49 |  | 646 | 50 |
|  | ≥50 |  | 231 | 18 |
| Marital status | | 1271 |  |  |
|  | Single |  | 140 | 11 |
|  | Married or long-term relationship |  | 1016 | 79 |
|  | Former relationship |  | 115 | 9 |
| Service | | 1279 |  |  |
|  | Naval Services |  | 161 | 12 |
|  | Army |  | 849 | 66 |
|  | Royal Air Force |  | 269 | 21 |
| Rank | | 1279 |  |  |
|  | Commissioned Officer |  | 332 | 25 |
|  | Non-commissioned Officer |  | 765 | 59 |
|  | Other Ranks |  | 182 | 14 |
| Engagement | | 1279 |  |  |
|  | Regular |  | 1023 | 79 |
|  | Reservist |  | 256 | 20 |
| Serving Status | | 1279 |  |  |
|  | Serving |  | 707 | 55 |
|  | Discharged |  | 572 | 44 |
| Deployment | | 1279 |  |  |
|  | Not Deployed |  | 319 | 24 |
|  | Afghanistan |  | 253 | 19 |
|  | Iraq |  | 315 | 24 |
|  | Both |  | 392 | 30 |
| Met Criteria for Probable Mental Health Disorder ^b^ | | 1278 |  |  |
|  | Anxiety |  | 228 | 17 |
|  | PTSD |  | 96 | 7 |
|  | Depression |  | 107 | 8 |
|  | Alcohol Misuse | 1279 | 236 | 18 |

^a^ Total personnel for which information was available; missing data where total is not 1279

^b^ General Anxiety Disorder (GAD 7) identifies probable anxiety; Patient Health Questionnaire (PHQ-9) identifies probable depression; The PTSD Checklist (PCL 5) identifies probable post-traumatic stress disorder; Alcohol Use Disorders Identification Test-C (AUDIT C) uses three questions to assess alcohol use.
